# Isoalantolactone restores the sensitivity of gram‐negative *Enterobacteriaceae* carrying MCR‐1 to carbapenems

**DOI:** 10.1111/jcmm.14936

**Published:** 2020-01-19

**Authors:** Na Lu, Qianghua Lv, Xiaodi Sun, Yonglin Zhou, Yan Guo, Jiazhang Qiu, Peng Zhang, Jianfeng Wang

**Affiliations:** ^1^ Department of Thoracic Surgery The First Hospital of Jilin University Changchun China; ^2^ Key Laboratory of Zoonosis Research Ministry of Education Institute of Zoonosis College of Veterinary Medicine Jilin University Changchun China

**Keywords:** isoalantolactone, *mcr‐1*, polycolistin B, synergistic effect

## Abstract

Polymyxin B has been re‐applied to the clinic as the final choice for the treatment of multidrug‐resistant gram‐negative pathogenic infections, but the use of polymyxin B has been re‐assessed because of the emergence and spread of the plasmid‐mediated *mcr‐1* gene. The purpose of this study was to search for an MCR inhibitor synergistically acting with polymyxin to treat the infection caused by this pathogen. In this study, we used the broth microdilution checkerboard method to evaluate the synergistic effect of isoalantolactone (IAL) and polymyxin B on *mcr‐1*‐positive *Enterobacteriaceae*. Growth curve analysis, time‐killing assays and a combined disc test were used to further verify the efficacy of the combined drug. Colonization of the thigh muscle in mice, survival experiments and lung tissue section observations was used to determine the effect of synergy in vivo after *Klebsiella pneumoniae* and *Escherichia coli* infection. We screened a natural compound, IAL, which can enhance the sensitivity of polymyxin B to *mcr‐1*‐positive *Enterobacteriaceae*. The results showed that the combined use of polymyxin B and IAL has a synergistic effect on *mcr‐1*‐positive *Enterobacteriaceae*, such as *K pneumoniae* and *E coli*, not only in vitro but also in vivo. Our results indicate that IAL is a natural compound with broad application prospects that can prolong the service life of polymyxin B and make outstanding contributions to the treatment of gram‐negative *Enterobacteriaceae* infections resistant to polymyxin B.

## 1 INTRODUCTION

Infections caused by multidrug‐resistant (MDR) gram‐negative pathogens have become one of the greatest threats to biohealth worldwide, leading to a huge challenge in clinical treatment.^1^ Multidrug‐resistant organisms such as *Klebsiella pneumoniae* and *E coli* have evolved multiple resistance mechanisms that limit treatment options.^2^ Polymyxins have good bactericidal effects against gram‐negative pathogens and are regarded as the last line of defence for the treatment of such bacterial pathogens.^3^, ^4^ Additionally, among these antibiotics, polymyxin B and colistin are cationic peptide antibiotics with lipophilic acyl side chains^5^ that were developed in the 1940s but fell into disfavour because of their high toxicity rates.^6^ The mechanism by which polymyxin kills gram‐negative pathogens relies on disrupting membrane permeability through polar and hydrophobic interactions. In these interactions, there is an electrostatic interaction between the positively charged residues of polymyxin and the negatively charged lipid A moiety of the lipopolysaccharide.^7^, ^8^ With the emergence of multidrug‐ and pandrug‐resistant gram‐negative pathogens in the 1990s, especially carbapenem‐resistant *Enterobacter*, we have to reactivate polymyxin in the absence of new antibacterials.^9^, ^10^, ^11^

Since the first discovery of the transferable colistin resistance gene *mcr‐1* in China, more than 40 countries and regions around the world have detected such resistance genes.^12^ MCR‐1, a protein product of the *mcr‐1* gene, is predicted to be an integral membrane protein with the catalytic activity of phosphoethanolamine transferases. The MCR‐1 enzyme modifies the chemical structure of the lipid A moiety on bacterial LPS by the addition of phosphoethanolamine, which in turn reduces the binding affinity of LPS to colistin.^13^ The *mcr‐1* gene can be spread between different bacteria in various regions by plasmids or transposition, which not only poses a great threat to public health but also poses a great challenge to clinical anti‐infective treatment.^14^ The emergence of the transferable polymyxin resistance genes *mcr‐1* represents a new mechanism of bacterial resistance that greatly challenges the last line of defence for the treatment of multidrug‐resistant gram‐negative pathogens.^15^

There is a need for an MCR inhibitor that works synergistically with polymyxin to treat infections caused by polymyxin B‐resistant *mcr‐1*‐positive *Enterobacteriaceae*. Isoalantolactone (IAL) has been shown to possess various pharmacological activities, including antitrypanosomal, anti‐apoptosis and antimicrobial activities.^16^, ^17^, ^18^ In addition, it has been reported that IAL has anticancer activity against several cancer cells, such as cervical squamous cell carcinoma, prostate cancer and gastric adenocarcinoma.^19^, ^20^ However, as a promising natural compound, the inhibitory effect of IAL against bacterial resistance enzymes has not been reported.

Here, we screened IAL as a new MCR‐1 inhibitor with the ability to enhance the sensitivity of *mcr‐1*‐positive *Enterobacteriaceae* to polymyxin B. Furthermore, the synergistic effect of IAL and polymyxin B was determined both in vivo and in vitro*.*

## 2 MATERIALS AND METHODS

### 2.1 Bacterial strains and reagents

The strains used in this experiment were described in our preceding studies^21^, ^22^, ^23^ and are listed in Table 1. Isoalantolactone (IAL) was purchased from Chengdu Ruifensi Biotechnology Co. Ltd. Colistin, polymyxin B and penicillin were purchased from the National Institute for the Control of Pharmaceutical and Biological Products (Beijing, China). Streptomycin sulphate, kanamycin sulphate, erythromycin and achromycin were purchased from Dalian Meilun Biotechnology Co Ltd. Stock solutions of IAL were prepared in dimethyl sulfoxide (Sigma‐Aldrich) at a concentration of 20 mg/mL.

**Table 1:** MIC values of the different antibiotics and ISO combination therapy for each of the tested bacterial isolates

Species	Source	*mcr‐1* confirmation	Antibiotics	MIC (μg/mL)		FIC index
				Alone	Combination	
*Escherichia coli* BL21(DE3) (pET28a‐*mcr‐1*)	Laboratory strain carrying the *mcr‐1* gene from *Klebsiella pneumoniae* ZJ05	+	Polymyxin B	10.00 ± 3.46	1.13 ± 0.54	**0.0.20 ± 0.08**
			Colistin	16.00 ± 0.00	2.00 ± 0.00	**0.19 ± 0.00**
*E coli* ZJ478	Human intra‐abdominal fluid	+	Polymyxin B	16.00 ± 11.31	4.67 ± 2.49	**0.40 ± 0.12**
			Colistin	8.00 ± 0.00	3.00 ± 1.41	**0.44 ± 0.18**
*E coli* DZ2‐12R	Chicken cloacae	+	Polymyxin B	7.33 ± 4.27	4.67 ± 1.49	**0.41 ± 0.12**
			Colistin	8.00 ± 0.00	3.00 ± 1.00	**0.44 ± 0.13**
			Ciprofloxacin	256.00 ± 0.00	256.00 ± 0.00	1.06 ± 0.00
			Kanamycin	256.00 ± 0.00	256.00 ± 0.00	1.06 ± 0.00
			Erythromycin	256.00 ± 0.00	256.00 ± 0.00	1.06 ± 0.00
			Penicillin	256.00 ± 0.00	256.00 ± 0.00	1.06 ± 0.00
			Tetracycline	128.00 ± 0.00	256.00 ± 0.00	2.06 ± 0.00
*K pneumoniae* ZJ02	Remote tertiary care hospital	+	Polymyxin B	64.00 ± 0.00	3.60 ± 0.80	**0.13 ± 0.03**
			Colistin	32.00 ± 0.00	6.00 ± 2.00	**0.25 ± 0.06**
			Ciprofloxacin	256.00 ± 0.00	256.00 ± 0.00	1.06 ± 0.00
			Kanamycin	256.00 ± 0.00	256.00 ± 0.00	1.06 ± 0.00
			Erythromycin	256.00 ± 0.00c	256.00 ± 0.00	1.06 ± 0.00
			Penicillin	256.00 ± 0.00	256.00 ± 0.00	1.06 ± 0.00
			Tetracycline	256.00 ± 0.00	256.00 ± 0.00	1.06 ± 0.00
*K pneumoniae* E831	Chicken cloacae	+	Polymyxin B	24.00 ± 8.00	6.00 ± 2.00	**0.38 ± 0.19**
			Colistin	32.00 ± 0.00	6.00 ± 2.00	**0.25 ± 0.06**
*K pneumoniae* ZJ05	Remote tertiary care hospital	+	Polymyxin B	40.00 ± 13.86	8.00 ± 4.90	**0.25 ± 0.06**
			Colistin	48.00 ± 16.00	3.00 ± 1.00	**0.13 ± 0.00**
*K pneumoniae* 13b5	Chicken cloacae	+	Polymyxin B	32.00 ± 0.00	8.00 ± 0.00	**0.31 ± 0.00**
			Colistin	32.00 ± 0.00	4.00 ± 0.00	**0.19 ± 0.00**
*K pneumoniae* K7	The People's Hospital of Jilin Province	−	Polymyxin B	1.38 ± 0.75	1.50 ± 0.58	1.69 ± 1.60
			Colistin	3.67 ± 3.79	1.00 ± 0.87	**0.40 ± 0.14**
*E coli* ATCC25922	ATCC	−	Polymyxin B	0.50 ± 0.00	0.50 ± 0.00	1.06 ± 0.00
			colistin	0.50 ± 0.00	0.50 ± 0.00	1.06 ± 0.00
*E coli* BL21(DE3) (pET28a)	Laboratory strain	−	Polymyxin B	0.75 ± 0.35	0.75 ± 0.35	1.06 ± 0.00
			colistin	0.50 ± 0.00	0.38 ± 0.18	0.81 ± 0.35

Note

The concentration of ISO was 32 μg/mL for all tested strains. All the MIC results were counted as no less than 3 times. The FICs lower than 0.5 was displayed in bold and recognized as synergistic effect.

### 2.2 FIC values determination

The microbroth checkerboard dilution method recommended by the Nation Committee for Clinical Laboratory Standards (NCCLS)^24^, ^25^ was employed to evaluate the combined bactericidal effect of IAL and polymyxin B. The strains listed in Table 1 were inoculated into 2 mL of LB medium overnight at 37°C with shaking at 200 rpm. The polymyxin B was diluted with LB medium for the antibiotic group without IAL, and the other group was combined with 32 μg/mL IAL. The results were observed after 24 hours of culture. The FIC values were determined as follows: FIC index = (FIC of polymyxin) + (FIC of IAL).^26^

### 2.3 Growth curve determination

The tested strains *K pneumoniae* ZJ02, *E coli* DZ2‐12R or *E coli* BL21(DE3) (pET28a‐*mcr‐1*) were inoculated into a 2 mL of LB overnight and expanded into 100 mL of LB medium. When the OD value of the bacterial solution reached 0.3, the LB medium was quantitatively dispensed and supplemented with IAL at final concentrations of 0, 16, 32, 64 or 128 μg/mL. The cultures were grown at 37°C with shaking at 200 rpm, and a growth curve was plotted by monitoring the OD_600nm_ value at 0.5‐ to 1‐hour intervals.

### 2.4 Time‐killing assays

The bactericidal curve was used to evaluate the potential bactericidal effect of IAL combined with polymyxin B. For the tested strains *K pneumoniae* ZJ02, *E coli* BL21(DE3)(PET28a‐*mcr‐1*) and *E coli* DZ2‐12R, the concentrations of polymyxin B used in this study were 4, 2 and 2 μg/mL, respectively. The concentration of IAL was 32 μg/mL. The tested strains were treated with IAL, polymyxin B, a combination of IAL and polymyxin B, or were not treated (positive control). The bacterial solution of each sample was diluted with PBS, plated on TSB solid medium and grown for 0, 1, 3, 5 and 7 hours. Following cultivation in a 37°C incubator for 24 hours, the bacteria on the plate were counted to construct the time‐killing curve.

### 2.5 Combined disc test

The bacterial solutions (MCR‐1‐positive *E coli* BL21(DE3) (pET28a‐*mcr‐1*), *E coli* DZ2‐12R and *K pneumoniae* ZJ02 or negative control bacteria, *E coli* ATCC 25922) were diluted 1:4000 and uniformly coated on the surface of LB solid medium containing various concentrations of IAL (0, 4 or 32 μg/mL). Then, a tablet containing polymyxin B was placed in the middle of the plate. The diameter of the inhibition zones was measured to evaluate the combination effect after being placed in a 37°C incubator for 24 hours.

### 2.6 Western blot assay

The tested strains *K pneumoniae* ZJ02, *E coli* DZ2‐12R or *E coli* BL21 (DE3) (pET28a‐*mcr‐1*) were cocultured with various concentrations of IAL (0, 4 and 32 μg/mL) as described in the growth curve determination for 4 hours. Following centrifugation at 13 800 *g* for 5 min, the precipitate of each sample was resuspended in loading buffer, mixed, boiled at 100°C for 7 minutes and separated by SDS‐PAGE.^27^ Then, the protein was transferred to a PVDF membrane. After blocking, the mouse monoclonal antibody against MCR‐1 (1:2000, Laboratory preservation), goat antimouse HRP‐conjugated secondary antibodies (1:2000, Proteintech) were applied for the determination of MCR‐1 production as described in our previous study.^23^ Isocitrate dehydrogenase (ICDH) was used as an internal control according to a previous study.

### 2.7 Ethics statement

Six‐ to eight‐week‐old female Balb/c mice with an average bodyweight of 20 ± 2 g were purchased from the Experimental Animal Center of Jilin University (Changchun, Jilin, China). Animal experiments were approved by and conducted in accordance with the guidelines of the Animal Care and Use Committee of Jilin University.

### 2.8 Mouse thigh infection with *K pneumoniae* ZJ02 and *E coli* DZ2‐12R

Establishment of a model of mouse thigh muscle infection by experimental methods in the literature.^28^ In this experiment, *K pneumoniae* ZJ02 or *E coli* DZ2‐12R was injected intramuscularly into the thigh muscle with 2 × 10^7^ CFUs per mouse. After inoculation, the mice were divided randomly into four groups (n = 5): (a) solvent (50 μL of DMSO), control treatment group; (b) IAL (50 mg/kg of bodyweight), alone treatment group; (c) polymyxin B (20 mg/kg), alone treatment group; and (d) a combination treatment group with IAL (50 mg/kg) and polymyxin B (20 mg/kg). Following simultaneous subcutaneous administration and subsequent administration every 8 hours, the infected mice of each group were killed at 36 hours post‐infection. The muscle tissue was taken, ground evenly, diluted with PBS and plated to determine the bacterial burden in the thigh muscle of infected mice.

### 2.9 Survival analysis

Overnight culture of *E coli* DZ2‐12R was injected intraperitoneally into mice with 2 × 10^8^ CFUs per mouse. After inoculation, the mice were divided randomly into four groups (n = 10): (a) solvent (50 μL of DMSO), control treatment group; (b), IAL (50 mg/kg of bodyweight), alone treatment group; (c) polymyxin B (5 mg/kg), alone treatment group; and (d) a combination treatment group with IAL (50 mg/kg) and polymyxin B (5 mg/kg). Following simultaneous subcutaneous administration and subsequent administration every 8 hours for 72 hours, the survival of infected mice was observed for 1 week.

### 2.10 Lung tissue section experiment

Overnight culture of *K pneumoniae* ZJ02 was injected transnasally into mice with 5 × 10^7^ CFUs per mouse. After inoculation, the mice were divided randomly into four groups (n = 5): (a) solvent (50 μL of DMSO), control treatment group; (b) IAL (50 mg/kg of bodyweight), alone treatment group; (c) polymyxin B (5 mg/kg), alone treatment group; and (d) a combination treatment group with IAL (50 mg/kg) and polymyxin B (5 mg/kg). Following simultaneous subcutaneous administration and subsequent administration every 8 hours, the infected mice of each group were killed at 72 hours post‐infection. The lung was taken, immersed in 4% formalin, stained with haematoxylin and eosin and visualized under a microscope.

### 2.11 Statistical analysis

The data are presented as the average plus the relative standard deviation and analysed with Student's *t* test. *P*‐value of <.05 was considered significant. *, *P* < .05; **, *P* < .01.

## 3 RESULTS

### 3.1 IAL enhances *mcr*‐*1‐*positive *Enterobacteriaceae* sensitivity to carbapenems without affecting bacterial growth

The checkerboard method is commonly used to screen inhibitors of resistance enzymes.^29^ Here, we found that 32 μg/mL IAL, combined with polymyxin B or colistin, had a significantly synergistic effect against the engineering strain *E coli* BL21(DE3) (pET28a‐*mcr‐1*) (Table 1). Consistent with this result, all FIC values were lower than 0.5 for the *mcr‐1*‐positive *E coli* strains (ZJ478 and DZ2‐12R) and *K pneumoniae* strains (ZJ02, E831, E831 and 13b5) following a combination treatment with IAL (32 μg/mL) and carbapenems (polymyxin B or colistin). The combination treatment with IAL and polymyxin B resulted in a 16‐fold reduction (from 64 to 4 μg/mL) in the MICs of *K pneumoniae* ZJ02, a clinical strain carrying *mcr‐1.* Although a synergistic effect was observed for the combination of IAL and colistin for *K pneumoniae* K7, the combined treatment with IAL and carbapenems (polymyxin B or colistin) showed no such synergistic effect for *mcr‐1*‐negative *E coli* strains (BL21(DE3) (pET28a) and ATCC25922) and *K pneumoniae* K7. Thus, these results indicated that IAL is an effective MCR‐1 inhibitor with the ability to restore the antibacterial activity of carbapenems (polymyxin B or colistin) against *mcr‐1*‐positive bacteria.

**Figure 1:**
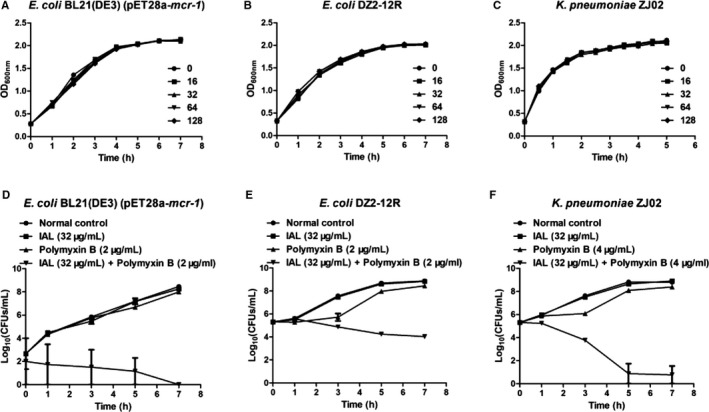
IAL restores the antibacterial activity of polymyxin B against MCR‐1‐positive bacteria without affecting the viability of the tested strains. The growth curves of *Escherichia coli* BL21(DE3) (pET28a‐*mcr‐1*) (A), *E coli* DZ2‐12R (B) and *Klebsiella pneumoniae* ZJ02 (C) within 7 h in the presence of different concentrations of IAL varying from 16 to 128 μg/mL. Time‐killing analysis with the indicated treatment for the *E coli* BL21(DE3) (pET28a‐*mcr‐1*) (D), *E coli* DZ2‐12R (E) and *K pneumoniae* ZJ02 (F)

**Figure 2:**
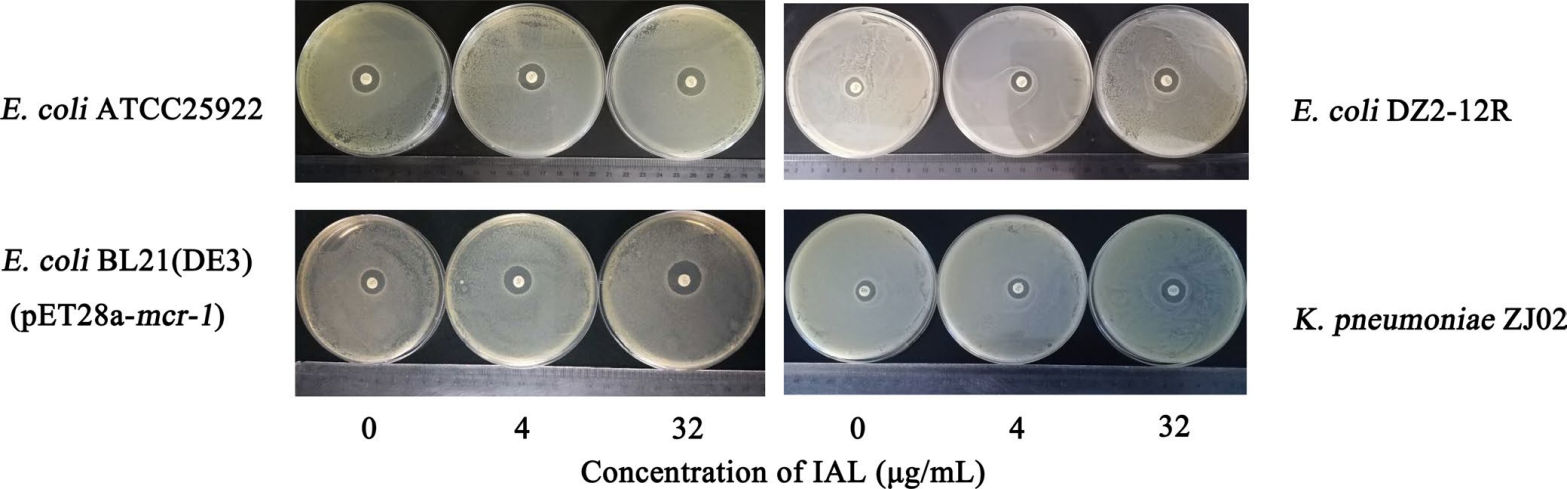
Zones of inhibition surrounding polymyxin B discs supplemented with IAL. MCR‐1‐positive bacteria (*Escherichia coli* BL21(DE3) (pET28a‐*mcr‐1*), *E coli* DZ2‐12R and *Klebsiella pneumoniae* ZJ02 and MCR‐1‐negative bacteria (*E coli* ATCC 25922) were coated on Luria Broth agar plates supplemented with the indicated concentrations of IAL (0, 4 and 32 µg/mL). Following incubation in a 37°C incubator for 24 h, the diameters of the inhibition zones were observed

**Figure 3:**
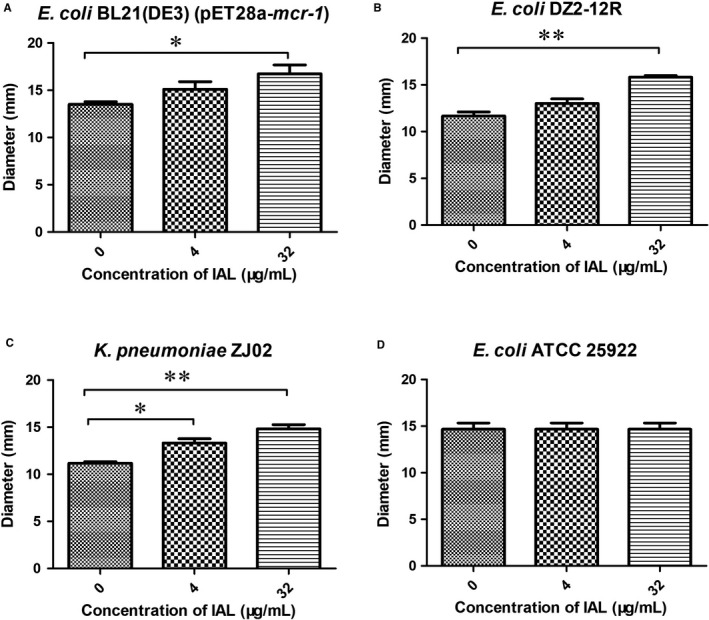
Combined disc tests for colistin in combination with IAL for each of the tested bacterial isolates. The diameter of the bacteriostatic region in Figure 2 was measured with a ruler. A, MCR‐1‐positive bacteria (*Escherichia coli* BL21(DE3) (pET28a‐*mcr‐1*). B, MCR‐1‐positive bacteria *E coli* DZ2‐12R. C, MCR‐1‐positive bacteria *Klebsiella pneumoniae* ZJ02. D, MCR‐1‐negative bacteria (*E coli* ATCC 25 922). *, *P* < .05; **, *P* < .01

However, the treatment with IAL alone at concentrations varying from 16 to 128 μg/mL had no effect on the growth of the tested strains (*E coli* BL21(DE3) (pET28a‐*mcr‐1*), *E coli* DZ2‐12R and *K pneumoniae* ZJ02, shown in Figure 1A‐C, respectively), suggesting that no antibacterial activity existed at IAL concentrations lower than 128 μg/mL. Furthermore, the synergistic effect of IAL and carbapenems was also observed in the time‐killing analysis (Figure 1D,F). For the tested strains, neither IAL nor polymyxin B has no bactericidal effect. The combination of polymyxin and IAL led to a bactericidal effect for *E coli* BL21 (DE3) (pET28a‐*mcr‐1*) and significant inhibition of bacterial growth for *E coli* DZ2‐12R and *K pneumonia* ZJ02 within 7 hours (Figure 1D‐F). In agreement with the above results, IAL combined with polymyxin significantly increased the inhibition zone from 11.17 ± 0.24 mm to 16.73 ± 1.32 mm for the *mcr‐1*‐positive strains *E coli* DZ2‐12R and *K pneumoniae* ZJ02 but not the *mcr‐1*‐negative strain *E coli* ATCC25922 (Figures 2 and 3). Taken together, our results established that IAL, as an effective MCR‐1 inhibitor with negative antibacterial activity, recovered the antibacterial activity of carbapenems against *mcr‐1*‐positive *Enterobacteriaceae*.

### 3.2 IAL had no inhibitory effect on MCR‐1 production

**Figure 4:**
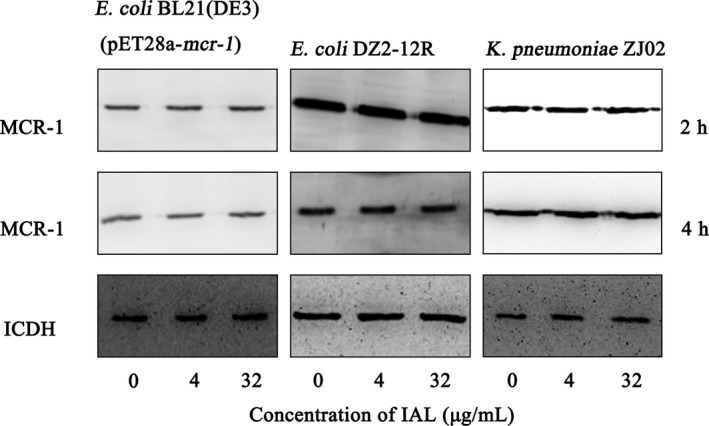
IAL has no influence on MCR‐1 expression. The MCR‐1‐positive bacteria *E coli* BL21(DE3) (pET28a‐*mcr‐1*), *Escherichia coli* DZ2‐12R or *K pneumoniae* ZJ02 were cultured with various concentrations of IAL (0, 4 and 32 µg/mL), and the production of MCR‐1 in cells was examined by Western blot analysis

A direct inhibition of MCR‐1 activity or MCR‐1 production may contribute to the synergistic effect of IAL with carbapenems. Western blot analysis was further employed to evaluate whether IAL treatment inhibits MCR‐1 production.^30^ As shown in Figure 4, the treatment with IAL at the concentrations required for the synergistic effect had no visible influence on the production of MCR‐1 by the tested strains. Thus, our results suggested that IAL effectively inhibits MCR‐1 without affecting the production of this enzyme protein.

### 3.3 Combination therapy of IAL and polymyxin B synergistically inhibits the pathogenicity of *mcr‐1*‐positive bacteria in vivo

**Figure 5:**
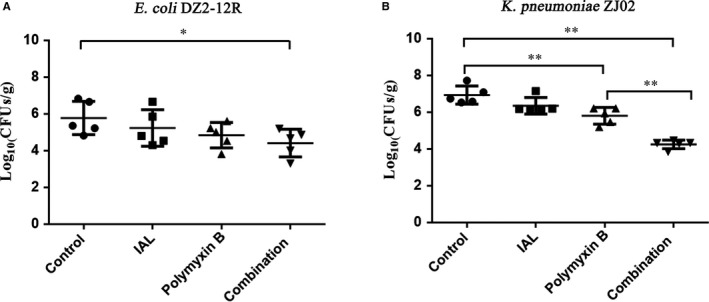
IAL combined with polymyxin B reduced the bacterial burden in the thigh muscle in mice infected with MCR‐1‐positive bacteria. Mice were intramuscularly infected with *Escherichia coli* DZ2‐12R (A) or *Klebsiella pneumoniae* ZJ02 (B) at a dose of 2 × 10^7^ CFUs per mouse. Following treatment with the solvent control (50 μL of DMSO), IAL (50 mg/kg), polymyxin B (20 mg/kg) or the combination therapy (50 mg/kg IAL + 20 mg/kg polymyxin B), the bacterial count in the thigh muscle of infected mice was examined by plating. **, *P* < .01; *, *P* < .05

To detect the same synergistic bactericidal effect of IAL and polymyxin B for *K pneumoniae* ZJ02 and *E coli* DZ2‐12R in vivo, a model of mouse thigh muscle infection was established in this study^31^ by assessing the bacterial burden in the thigh 36 hours post‐infection. As shown in Figure 5A, treatment with IAL or polymyxin B alone did not result in an evident reduction in the bacterial burden in *E coli* DZ2‐12R‐infected mice. However, a combination therapy of IAL and polymyxin B significantly reduced the bacterial colonization in the thigh (Figure 5A). Although a significant difference between the polymyxin B treatment and solvent control was observed for the *K pneumoniae* ZJ02‐infected mice, the combined therapy resulted in a more than 1.5‐log_10_ reduction of the bacterial burden in infected mice when compared with the polymyxin B treatment (Figure 5B). Together, these results indicated that IAL combined with polymyxin B synergistically inhibited the bacterial burden in mice.

### 3.4 Protection rate experiment

**Figure 6:**
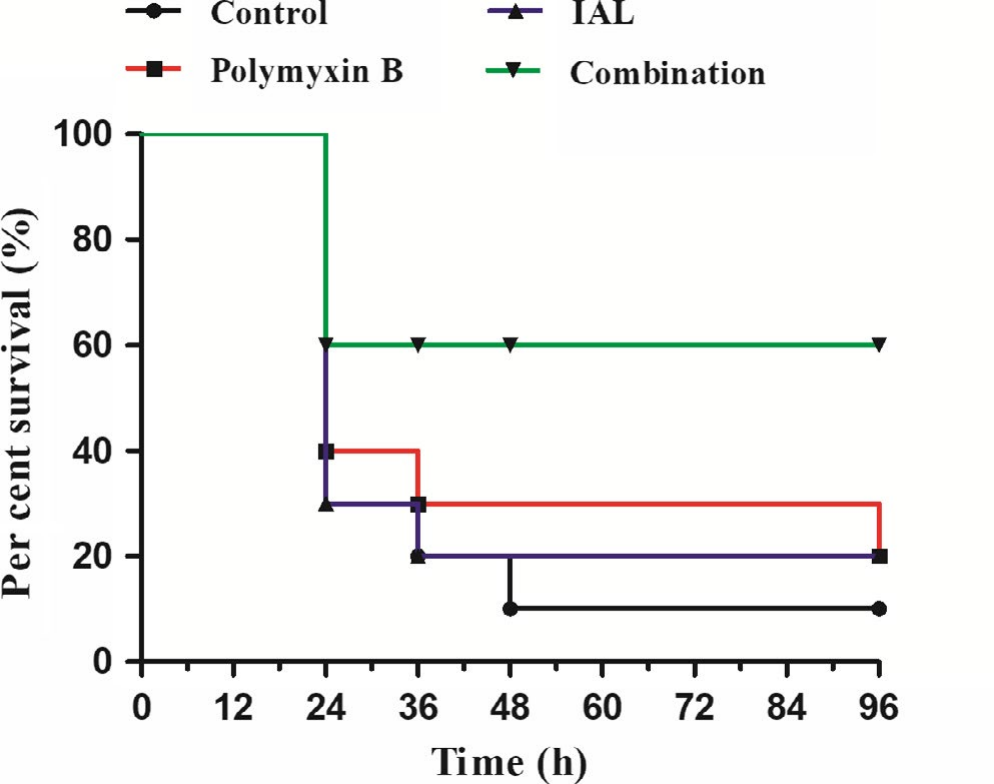
IAL combined with polymyxin B increased the survival rate of *Escherichia coli* DZ2‐12R in infected mice. Each mouse was intraperitoneally injected with 2 × 10^8^ CFUs *E coli* DZ2‐12R and treated with the solvent control (50 μL or DMSO), IAL (50 mg/kg), polymyxin B (5 mg/kg) or the combination therapy (50 mg/kg IAL + 5 mg/kg polymyxin B) for 72 h. The survival of infected mice was observed after 1 wk

To further evaluate this synergistic effect, the survival rate of *E coli* DZ2‐12R in intraperitoneally infected mice treated with IAL, polymyxin B or a combination of IAL and polymyxin B was monitored for 72 hours. As expected, all infected mice were dead after 72 hours. After infection, the mice were monitored for 3 days using the same treatment method, combined with the solvent control group and the IAL group alone, and the survival rate of the mice in the antibiotic group alone was 20% (Figure 6). The combined polymyxin B and IAL group had a survival rate of 60% (Figure 6), further confirming that the combination treatment has a better effect than the drug or antibiotic alone.

**Figure 7:**
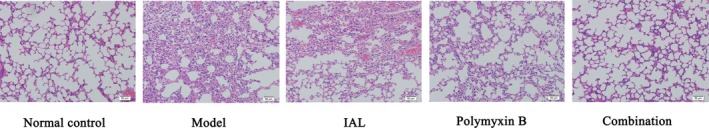
IAL combined with polymyxin B alleviated the pathogenic injury in the lungs of *Klebsiella pneumoniae* ZJ02‐infected mice. Each mouse was transnasally infected with 5 × 10^7^ CFUs *K pneumoniae* ZJ02 and treated with the solvent control (50 μL of DMSO), IAL (50 mg/kg), polymyxin B (5 mg/kg) or the combination therapy (50 mg/kg IAL + 5 mg/kg polymyxin B). Then, the lungs of infected mice were analysed by histopathological analysis

Finally, the pathological damage in the lung was assessed in a mouse model of pneumonia by *K pneumoniae* ZJ02. As shown in Figure 7, no pathological damage was observed for the uninfected mice under the microscope. In the infected mice, the alveolar cavity was filled with exuded cellulose, alveolar septum thickening and capillaries. Consistent with the above results, the symptoms of the drug‐treated group and the antibiotic‐treated group were slightly relieved (Figure 7). However, the cellulose exudation in the alveolar cavity was reduced, and the pulmonary interstitial was only infiltrated by a small amount of inflammatory cells, suggesting that the pathological damage was significantly reduced (Figure 7). Taken together, IAL combined with polymyxin B remarkably inhibited the pathogenicity of *mcr‐1*‐positive bacteria in mice.

## 4 DISCUSSION

The emergence and spread of drug‐resistant bacteria that have caused serious clinical infections is a matter of great concern to researchers. The prevalence of *mcr‐1*‐positive *K pneumoniae* and *E coli* has brought great challenges to clinical treatment, which calls for novel strategies or agents to fight these bacterial pathogens. Among these strategies, the identification of effective MCR‐1 inhibitors combined with antibiotics is a promising strategy to address this challenge. Here, we successfully screened the natural compound IAL as an effective agent with the capability of restoring the antibacterial activity of carbapenems against *mcr‐1*‐positive *Enterobacteriaceae*. These results suggest that IAL is a potential leading compound for the treatment of *mcr‐1*‐positive bacteria when combined with antibiotics.

Isoalantolactone (IAL), the main sesquiterpene lactone in Radix Inulae and other plants, is a frequently utilized herbal medicine. Isoalantolactone has various pharmacologic effects,^31^, ^32^ such as inhibiting inflammation, preventing proliferation and inducing apoptosis.^33^ Furthermore, this compound has been reported to significantly induce breast cancer cell apoptosis by activating the caspase cascade, cleaving poly (ADP‐ribose) polymerase.^34^ In addition, IAL was recently identified as selectively toxic to cancer cells.^18^, ^35^ These results suggested that IAL is a multiple biological compound, as a drug, used in clinical practice. However, IAL is not soluble in water, which limited its pharmacological activity in vitro/in vivo. Therefore, the molecular structure of IAL requires further modification.

In the present study, we confirmed the synergistic effect of polymyxin B and IAL on plasmid‐mediated *mcr‐1*‐positive *Enterobacteriaceae*, both in vitro and in vivo. From checkerboard microdilution assays, we found that IAL only has a synergistic effect on *mcr‐1*‐positive *Enterobacteriaceae* in combination with carbapenem antibiotics, but it has no effect when used in combination with other antibiotics. Moreover, IAL has no synergistic effect on *mcr‐1*‐negative isolates (Table 1). Additionally, IAL treatment did not affect the production of MCR‐1 in *mcr‐1*‐positive *Enterobacteriaceae* (Figure 4)*.* This particular synergistic effect suggests that IAL may act directly on MCR‐1 and affect its activity. Furthermore, the combination of isoalantolactone and polymyxin B had the best synergistic effect on *Klebsiella pneumonia*, with a 16‐fold change in the MIC values (Table 1). Upon examination, compared with other MCR‐1 inhibitors, IAL has a great advantage with a good synergistic effect on polymyxin‐resistant gram‐negative bacteria both in vitro and in vivo. All the information described suggested that IAL possesses the possibility for development into a pharmaceutical preparation for clinical use.

Some clinical drugs are often limited in their use because of poor absorption in vivo. However, in this study, subcutaneous administration of IAL and polymyxin B achieved good therapeutic effects for the animal infection treatment experiments. Our data indicated that absorption may not affect the usage of IAL as a drug.

Taken together, our results suggest that the combination of IAL and carbapenems can be an option for the treatment of MCR‐1‐positive *Enterobacteriaceae*. As a promising natural compound, IAL has more pharmacological activities to be discovered.

## CONFLICT OF INTEREST

The authors have no conflict of interest to declare.

## AUTHOR CONTRIBUTIONS

LN, LQ, SX, ZY, ZP and WJ conceived and performed all the experiments. LN, LQ and WJ researched the data contributed to the statistical analysis and discussion. QJ contributed to the discussion and reviewed the manuscript. All authors reviewed the manuscript.

## Data Availability

The data used to support the findings of this study are available from the corresponding author upon request.
